# Single- versus two-stage revision surgery in the case of fracture-related infection: a systematic review

**DOI:** 10.5194/jbji-10-347-2025

**Published:** 2025-10-01

**Authors:** Jonathan Sliepen, Michelle A. S. Buijs, Jolien Onsea, Geertje A. M. Govaert, Frank F. A. IJpma, Jean-Paul P. M. de Vries, Bart C. H. Van der Wal, Charalampos Zalavras, Willem-Jan Metsemakers

**Affiliations:** 1 Department of Trauma Surgery, University Medical Center Groningen, Groningen, the Netherlands; 2 Department of Trauma Surgery, University Hospitals Leuven, Leuven, Belgium; 3 Department of Trauma Surgery, University of Utrecht, University Medical Center Utrecht, Utrecht, the Netherlands; 4 Department of Development and Regeneration, KU Leuven – University of Leuven, Leuven, Belgium; 5 Department of Surgery, Division of Vascular Surgery, University Medical Center Groningen, University of Groningen, Groningen, the Netherlands; 6 Department of Orthopedic Surgery, University Medical Center Utrecht, Utrecht, the Netherlands; 7 Department of Orthopaedic Surgery, Los Angeles General Medical Center, Keck School of Medicine, University of Southern California, Los Angeles, USA

## Abstract

**Background**: This systematic review aimed to evaluate the current evidence regarding the clinical outcome of single- and two-stage revision procedures for long-bone fracture-related infection (FRI). The review focused on unhealed fractures without critical-sized bone defects, treated with internal fixation. **Methods**: A systematic review was conducted according to the Preferred Reporting Items for Systematic Reviews and Meta-Analyses (PRISMA) reporting guidelines. A systematic search was carried out in PubMed, Embase via Elsevier, and Web of Science Core collection. **Results**: Out of 21 126 articles initially identified, 35 studies, including 985 patients, were eligible for the final analysis. A total of 27 studies assessed single-stage revisions, 5 examined two-stage procedures, and 3 included both approaches. The mean bone-healing rate was 80 % for single-stage approaches and 77 % for two-stage approaches. The mean infection eradication rate for single-stage revisions was 87 %, whereas two-stage revisions demonstrated a mean infection eradication rate of 81 %. Only five studies included patients ( n= 34) diagnosed with an FRI within 6 months after the primary fracture fixation. **Conclusion**: For patients with unhealed long-bone FRIs without critical-sized bone defects, the current literature is of poor quality, heterogeneous, and lacks strong evidence to recommend either a single-stage or two-stage approach with internal fixation. For both protocols, the rate of revision surgery remains high. Furthermore, high-quality studies focusing on two-stage procedures, especially for the treatment of FRIs occurring within 6 months after initial fracture fixation, are almost non-existent. The identification of positive cultures during single-stage procedures for presumed aseptic fracture non-unions might be linked to poorer clinical outcomes.

## 1 Introduction

Despite advances in the diagnosis and management of fracture-related infection (FRI), this complication remains a complex and often difficult-to-treat disease entity, which poses a huge burden on patients, treating physicians, and healthcare systems (Metsemakers et al., 2024; Buijs et al., 2024; Iliaens et al., 2021). Currently, selecting the most suitable surgical treatment strategy can be challenging and depends on various aspects related to the fracture, patient, and soft tissue status (Alt et al., 2024).

The surgical management of FRI is based on two main strategies. The first consists of debridement, antimicrobial therapy, and implant retention (DAIR). The second consists of debridement; antimicrobial therapy; and either implant removal, if the fracture has healed, or exchange, if the fracture has not healed (Depypere et al., 2020). In case of unhealed fractures, implant exchange can be done in a single- or a two-stage procedure. In a single-stage approach, the infected hardware is removed, the infected area is thoroughly debrided, and new hardware is implanted in a single surgical session. This approach has the advantage of reducing the number of surgeries, potentially shortening the overall treatment duration and reducing healthcare costs. Conversely, a two-stage revision approach involves an initial surgery to remove the infected hardware, perform debridement, and provisionally stabilize the fracture with internal or external fixation. A second procedure is then conducted to remove the temporary fixation and implant new definitive hardware. This method facilitates, for example, a brief initial surgery in cases involving compromised hosts such as those with sepsis, enabling systemic and local antibiotic administration prior to definitive hardware implantation. Additionally, this approach is warranted in instances of severe soft tissue damage and the unavailability of a plastic and reconstructive surgeon.

To date, scientific data comparing single- and two-stage surgical revision procedures for FRI are scarce (Struijs et al., 2007). This is especially true for FRIs diagnosed within 6 months after fracture fixation. Furthermore, a multitude of distinct single- and two-stage FRI treatment strategies have been described, resulting in a heterogeneous group varying in terms of type of fracture fixation (i.e., external fixation or internal fixation), bone defect management (i.e., autologous bone grafts, distraction osteogenesis), and the use of local antimicrobials, which complicates comparison. In 2024, a systematic review and meta-analysis was published on the treatment results of single- and two-stage treatment of chronic osteomyelitis (Lari et al., 2024). However, the authors focused on healed fractures and other scenarios outside of the scope of this review.

The objective of this systematic review was to answer the following research questions:

- Is there sufficient evidence in the literature to substantiate the use of a single-stage versus a two-stage approach for the treatment of unhealed long-bone FRIs without critical-sized bone defects, utilizing internal fixation, with regards to bone healing and infection eradication?
- Is there scientific data solely focusing on single- and two-stage implant exchange for FRI occurring within 6 months after the primary fracture fixation?

The review specifically focused on unhealed fractures without critical-sized bone defects (as defined by Schemitsch, 2017), definitively treated with internal fixation.

## 2 Materials and methods

This systematic review was conducted according to the Preferred Reporting Items for Systematic Reviews and Meta-Analyses (PRISMA) reporting guidelines (Tricco et al., 2018) and is registered in the Prospero database (ID no. CRD42022355665).

### 2.1 Eligibility criteria

Studies were included if they fulfilled all of the following criteria: (1) reports on at least 10 long-bone (i.e., femur, tibia, humerus) human FRI cases in the presence of an unhealed fracture without a critical-sized bone defect and (2) provides a clear surgical protocol describing the surgical approach (single- or two-stage) and utilizing internal fixation for definitive refixation of the fracture. Studies were excluded if they focused on FRIs in healed fractures; FRIs associated with bone defects; FRIs in anatomical locations other than the femur, tibia, or humerus; FRIs in pathological fractures; FRIs in skeletally immature patients; non-traumatic osteomyelitis (e.g., hematogenous disseminated osteomyelitis); periprosthetic joint infections (PJIs); and FRIs managed by soft tissue reconstruction only. Furthermore, studies were excluded if they used external fixation as a definitive fracture fixation, if they did not report original data, or if FRI management was done with a multiple-stage approach.

### 2.2 Search strategy

Databases used for the search were PubMed, Embase via Elsevier, and Web of Science Core collection. Searches were run on 21 November 2024 (see Table S1 in the Supplement). Trial registries used for the search were the World Health Organization – International Clinical Trials Registry Platform (ICTRP) and Cochrane CENTRAL. They were searched on 21 November 2024 (see Table S1). This search string was constructed by two authors (Jonathan Sliepen and Willem-Jan Metsemakers) together with a biomedical reference librarian. Conference abstracts and pre-prints were excluded from the search. No restrictions on language or publication date were applied. Additionally, the search string was validated by checking whether it contained articles identified by supplementary searches prior to executing the search string. Results were de-duplicated in a stepwise manner using EndNote^™^ version 20 and were uploaded onto Rayyan (Ouzzani et al., 2016).

### 2.3 Study screening

**Figure 1:**
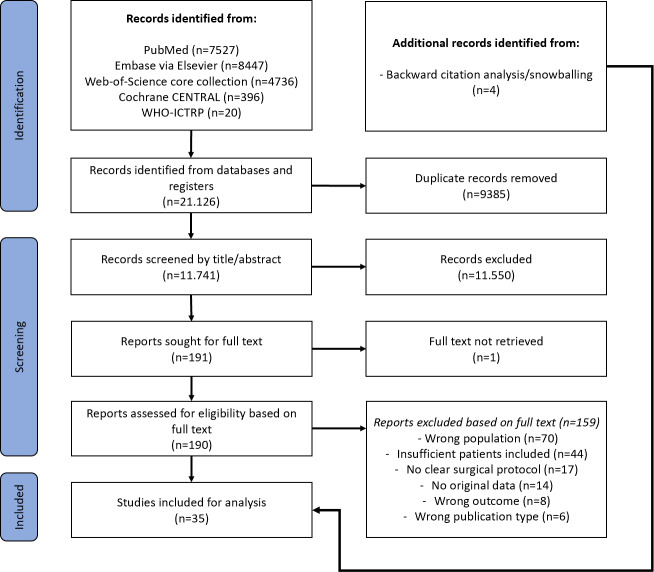
PRISMA flow diagram.

Search results were screened for eligibility by two authors (Jonathan Sliepen and Michelle A. S. Buijs) independently using Rayyan software (Ouzzani et al., 2016). Once the initial title and abstract screening was completed, the full text of the included studies was retrieved. When the full text was not available the corresponding author was contacted once via email. In cases where no response was received, the article was excluded. The full-text articles retrieved were subjected to a review process conducted by two independent authors (Jonathan Sliepen and Michelle A. S. Buijs) to ascertain their suitability for inclusion. Discrepancies were resolved by consensus or by referring to a third and fourth author (Willem-Jan Metsemakers and Charalampos Zalavras). The screening and inclusion process is shown in Fig. 1. A backward citation analysis was performed on all included articles to ensure the capturing of all available relevant literature.

### 2.4 Data extraction

We used a data extraction form for study characteristics and outcome data, which was tested on three studies in the review. Three study authors (Jonathan Sliepen, Willem-Jan Metsemakers, and Charalampos Zalavras) extracted the following data from the included studies: study types (randomized controlled trials, observational studies (all types), cohort (longitudinal) studies, case-control studies, before–after studies, and cross-sectional studies), methods (study authors, year, country of publication, and duration of follow-up), participants (number of participants relevant to this study), interventions (type of surgery, type of internal fixation, used local antimicrobials, soft tissue management, and complications), and outcomes (infection eradication rate (primary outcome), bone-healing rate (primary outcome), and the number of patients in whom secondary surgeries were performed). Clinical outcome was assessed by determining the bone-healing and infection eradication rate. The weighted mean of the bone-healing and infection eradication rate was calculated based on all studies describing the outcome. Secondary procedures were defined as surgical interventions performed to achieve infection eradication and/or bone healing, as described by the authors. Surgical interventions to remove implants after fracture healing and in the absence of infection were not regarded to be secondary procedures.

In order to establish more homogenous groups for the purpose of comparison, a number of subgroups were created based on all included studies (single- and two-stage). The first group consisted of studies that used a conventional implant without an additional antimicrobial coating as a revision implant. The second group consisted of studies which treated patients with custom-made polymethyl methacrylate (PMMA)-coated implants. The latter group contained studies with a significant difference in terms of the surgical technique, namely studies in which patients were treated with a conventional implant, such as intramedullary nails or plates that can be locked with screws, with a PMMA coating (group 2a), and studies which treated patients with non-conventional implants, such as K wires, guide wires, and threaded Ilizarov rods (which cannot be locked with screws), with a PMMA coating (group 2b). In addition, for studies focusing on a single-stage approach, we created a third group, comprising studies on the treatment of presumed aseptic fracture non-unions. These studies included patients without clinical and microbiological confirmatory and/or suggestive signs of infection. Only patients with positive cultures which were not considered to be contaminants and for whom there was the intention to treat the infection were included for this review. The data will be presented according to these subgroups throughout this paper.

### 2.5 Methodological quality assessment

The methodological quality and risk of bias of the included studies were independently assessed in accordance with to the guidelines of the McMaster University Occupational Therapy Evidence-Based Practice Research Group (Law et al., 1998). The assessment was conducted by two independent reviewers (Jonathan Sliepen and Michelle A. S. Buijs). Disagreements were resolved by consensus or by referring to a third and fourth author (Willem-Jan Metsemakers and Charalampos Zalavras). The McMaster critical appraisal consists of eight components: (1) study purpose, (2) literature review, (3) study design, (4) sample, (5) outcomes, (6) intervention, (7) results, and (8) conclusions and implications. All items had to be scored with “yes” = 1 point, “no” = 0 points, or NA (not applicable). The total score is the sum of all item scores, and this reflects the methodological quality of the study. The maximum score possible is 16 for randomized studies and 14 for other study designs. In cases where items cannot be scored due to irrelevance, these items should be subtracted from the maximum score. The final score may vary between 0 % and 100 %, with a higher score reflecting a higher methodological quality. Scores between 90 % and 100 % are indicative of excellent-quality studies, scores between 75 % and 89 % are indicative of good-quality studies, scores between 50 % and 74 % are indicative of moderate-quality studies, and scores below 50 % indicate poor-quality studies.

## 3 Results

### 3.1 Search and study characteristics

A total of 21 126 articles were identified on initial screening (Fig. 1). After removing duplicates, 11 741 articles remained. Of these, 191 articles were selected based on their title and abstract. The full text of all articles, except for 1, could be retrieved, and after selection, 32 articles were included. There were 3 additional articles included based on backward citation analysis and snowballing, resulting in a total of 35 included studies, with 985 patients. Table 1 provides an overview of all included studies. Out of the 35 included articles, 25 were retrospective studies, and 10 were prospective studies (Table 1). Six studies focused solely on FRI treatment of the femur, five studies focused solely on the tibia, but most studies ( n= 23) included patients with an FRI of the femur or tibia and/or humerus. Three studies did not specify which long bone was involved. A total of 12 studies had a follow-up of at least 12 months. A total of 11 studies had a follow-up of less than 12 months, and in 12 studies, the minimum duration of follow-up was not specified.

### 3.2 Methodological quality and risk of bias assessment

There were 1 randomized prospective study, 14 retrospective cohort studies, and 20 retrospective and/or prospective case series included in the analysis. Based on the McMaster University Occupational Therapy Evidence-Based Practice Research Group quality assessment (Law et al., 1998), the mean overall methodological quality score was 62 % for studies focused on single-stage revision and 68 % for studies focused on two-stage revision, indicating that the studies were of moderate methodological quality. For studies using conventional implants, the mean overall score was 74 %, whereas for studies which used custom-made PMMA-coated implants, the mean overall score was 56 % (moderate quality). The mean overall score for studies focused on presumed aseptic fracture non-unions was 90 % (excellent quality). The methodological quality was classified as poor in 11 studies, moderate in 13 studies, good in 7 studies, and excellent in 4 studies. The results of the methodological quality assessment are presented in Table S2.

**Table 1:** Overview of included studies.

Author(s), publication date	Study design	Type of surgery	N^1^	Anatomical location	Follow-up in months Mean (range)	Outcome (as defined in the article)		Complications
						Bone healing	Infection eradication	Secondary procedure(s)
Studies on presumed aseptic fracture non-union^19^								
Amorosa et al. (2013)^2^	Retrospective cohort study	Single-stage	19	Humerus ( n= 4), femur ( n= 8), tibia ( n= 7)	11 (2–45)	12/19; 63 %	Not specified	7/19; 37 %
Arsoy et al. (2018)^2^	Retrospective cohort study	Single-stage	41	Humerus ( n= 4), femur ( n= 20), tibia ( n= 17)	30 (4–136)^3^	30/41; 73 %	47/51; 92 %^4^	11/41; 27 %
Hackl et al. (2024)	Retrospective cohort study	Single-stage	25	Femur ( n= 25)	Not specified (minimum: 12)	14/25; 56 %	14/25; 56 %	11/25; 43 %
Wagner et al. (2024b)	Retrospective cohort study	Single-stage	14	Not specified	Not specified	13/14; 93 %	Not specified	1/14; 7 %
Studies with confirmed or suspected infection								
Prasarn et al. (2009)^2^	Case series	Single-stage	11	Femur ( n= 11)	67 (24–144)	10/11; 91 %	11/11; 100 %	7/11; 64 %
Amorosa et al. (2014)^2^	Retrospective cohort study	Single-stage	21	Femur ( n= 21)	Not specified	10/21; 48 %	18/21; 86 %	11/21; 52 %
Klemm (1986)^5^	Case series	Single-stage	64	Femur ( n= 37), tibia ( n= 27)	Not specified	53/64; 83 %	59/64; 92 %	6/64; 9 %
Shah et al. (2009)^5^	Case series	Single-stage	235	Femur, tibia ( n= 235)^6^	Not specified (minimum: 12)	213/250; 85 %^7^	213/250; 85 %^8^	150/250; 60 %^8^
Unsworth et al. (2024)	Retrospective cohort study	Single-stage	14	Not specified	Not specified (minimum: 12)	Not specified	230/252; 91 %^7^	19/252; 8 %^7^
Wu and Chen (2003)^9^	Case series	Single-stage	12	Femur ( n= 12)	Median: 48 (24–72)	11/12; 92 %	11/12; 92 %	1/12; 8 %
Babhulkar et al. (2005)^5^	Retrospective cohort study	Single-stage ( n= 8) Two-stage ( n= 9)	17	Femur ( n= 6), tibia ( n= 2), humerus ( n= 2) Femur ( n= 5), tibia ( n= 2)	40 (24–180)^3^	Single-stage 8/8; 100 % Two-stage 9/9; 100 %	Single stage 8/8; 100 % Two-stage 9/9; 100 %	Not specified
Wu et al. (2007)	Retrospective cohort study	Two-stage	15	Femur ( n= 15)	55 (24–156)	5/15; 33 %	5/15; 33 %	10/15; 67 %
Custom-made PMMA-coated implant studies^20^								
Bakshi et al. (2022)^10^	Randomized prospective cohort study	Single-stage	13	Not specified	Not specified	13/13; 100 %	16/20; 80 %^11^	Not specified
Bhatia et al. (2017)^10,12^	Case series	Single-stage	20	Tibia ( n= 20)	Not specified (mean: 13)	12/20; 60 %	12/20; 60 %	6/20; 30 %
Bidolegui et al. (2023)^10^	Retrospective cohort study	Single-stage	12	Femur, tibia ( n= 12)	18 (8–37)	9/12; 80 %	9/12; 80 %	12/12; 100%
Charan et al. (2023)^5,10,12^	Case series	Single-stage	15	Femur ( n= 3), tibia ( n= 12)	Not specified (mean: 13)	10/15; 67 %	13/15; 87 %	2/15; 13 %
Chavan et al. (2019)^5,10,12^	Case series	Single-stage	12	Femur ( n= 2), tibia ( n= 10)	6 (5–7)	8/12; 67 %	10/12; 83 %	4/12; 33 %
Conway et al. (2014)^5,10,^	Retrospective cohort study	Single-stage	21	Femur, tibia ( n= 21)^6^	39 (12–115)^3^	20/21; 95 %	21/21; 100 %	1/21; 5 %
Dar et al. (2017)^12^	Case series	Single-stage	11	Femur ( n= 7), tibia ( n= 4)	Not specified	10/11; 91 %	Not specified	1/11; 9 %
Gao et al. (2019)^10,12^	Case series	Single-stage	13	Humerus ( n= 1), femur ( n= 6), tibia ( n= 6)	20 (2–39)	10/13; 77 %	13/13; 100 %	2/13; 15 %
Jokhio et al. (2021)^10,12^	Case series	Single-stage	30	Tibia ( n= 30)	Not specified (mean: 13)	24/30; 80 %	28/30; 93 %	6/30; 20 %
Manikumar et al. (2024)^5,10^	Case series	Single-stage	14	Femur and tibia ( n= 14)	≥24	13/14; 93 %	12/14; 86 %	Not specified
Pradhan et al. (2017)^10^	Case series	Single-stage	21	Femur ( n= 21)	20 (14–28)	18/21; 86 %	19/21; 90 %	5/21; 24 %
Reddy et al. (2023)^5,10^	Retrospective cohort study	Single-stage	21	Femur and tibia ( n= 21)	Not specified	13/21; 62 %	21/21; 100 %	13/27; 48 %^13^
Rice et al. (2021)^10^	Case series	Single-stage	32	Humerus ( n= 1), femur ( n= 8), tibia ( n= 23)	Not specified	22/32; 69 %	22/32; 69 %	10/32; 31 %
Saravanan et al. (2017)^10^	Case series	Single-stage	25	Femur ( n= 6), tibia ( n= 19)	8 (8–36)^14^	20/25; 80 %	24/25; 96 %	3/25; 12 %

**Table 1:** Continued.

Author(s), publication date	Study design	Type of surgery	N^1^	Anatomical location	Follow-up in months Mean (range)	Outcome (as defined in the article)		Complications
						Bone healing	Infection eradication	Secondary procedure(s)
Selhi et al. (2012)	Case series	Single-stage	16	Humerus ( n= 1), femur ( n= 8), tibia ( n= 7)	Not specified	11/16; 69 %	11/16; 69 %	3/16; 19 %
Solanki et al. (2023)^10,12^	Case series	Single-stage	30	Femur ( n= 8), tibia ( n= 22)	8.2 (9–38)^14^	27/30; 90 %	28/30; 93 %	9/30; 30 %
Thonse and Conway (2008)^5,10,15^	Retrospective cohort study	Single-stage	34	Not specified	Group 1: 22.5 (2–60) Group 2: 4.5 (1–12)	41/49; 84 %	44/52; 85 %	14/52 (27 %)
Kumar and Shankar (2023)^5,10^	Case series	Single stage ( n= 16) Two-stage ( n= 4)	20	Femur and tibia ( n= 18)	10 (8–12)	15/20; 75 %^16^	16/20; 80 %^16^	7/20; 35 %^16^
Zalikha et al. (2023)^10^	Retrospective cohort study	Single-stage ( n= 25) Two-stage ( n= 16)	41	Humerus ( n= 1), femur ( n= 27), tibia ( n= 13)	44 (minimum: 24)	35/41; 85 %	35/41; 85 %	6/41; 15 %
Klemm and Hess (1995)	Case series	Two-stage	25	Tibia ( n= 25)	Not specified	22/25; 88 %	22/25; 88 %	3/25; 12 %
Qiang et al. (2007a)^5,12,17^	Case series	Two-stage	18	Femur ( n= 5), tibia ( n= 13)	16 (6–28)	11/18; 61 %	17/18; 94 %	Not specified
Reilly et al. (2016)	Retrospective cohort study	Two-stage	41	Tibia ( n= 41)	20 (6–76)	31/41; 76 %	31/41; 76 %	10/41; 24 %
Wang (2011)^18^	Case series	Two-stage	12	Femur ( n= 5), tibia ( n= 7)	34 (24–48)	12/12; 100 %	12/12; 100 %	Not specified

^1^ Number of patients included relevant for this study. ^2^ Possible overlapping populations with Amorosa et al. (2013) and Arsoy et al. (2018). ^3^ Duration of follow-up of patients within the scope of this study was not provided explicitly; duration of follow-up is based on the entire population or the subpopulation most closely related to the patients relevant to our study. ^4^ Arsoy et al. (2018) included 10 patients with an FRI at an anatomical location not of interest for this study. Four out of nine patients who underwent removal of osteosynthesis materials had positive cultures; it is unclear if these were patients with an FRI at an anatomical location of interest. ^5^ The numbers described in the text or tables do not match or add up. ^6^ The exact distribution is unclear due to the exclusion of patients beyond the scope of this study (e.g., with critical-sized bone defect, pathological fracture, anatomical location not of interest). ^7^ Only the outcomes of the entire cohort were described. It was not possible to extract the results of the patients relevant to this study. Hence, the results of the entire included cohort were reported (Shah et al., 2009; Unsworth et al., 2024). ^8^ In this study, 15 patients had a pathological fracture; it was not possible to extract their results separately. A total of 60 % of patients required additional surgeries, and so the success rate in this series after a single-stage is likely to be lower than 80 % (Shah et al., 2009). ^9^ In this study, no IV antimicrobials were started as local antibiotics covered all cultured pathogens. Systemic antimicrobial treatment was not described (Wu and Chen, 2003). ^10^ Routine removal of the implant was not described. ^11^ The infection eradication rate was not specified for the 13 relevant patients with a small bone defect (Bakshi et al., 2022). ^12^ A cast or brace was applied for fixation until union. ^13^ This study included six patients diagnosed with chronic osteomyelitis. The number of additional procedures undergone by patients relevant to this study was not specified. Hence, the results of the entire included cohort were reported (Reddy et al., 2023). ^14^ Error in this study: the mean duration of follow-up cannot be the smaller than or equal to the minimum value of the range (Saravanan et al., 2017; Solanki et al., 2023). ^15^ This study included 17 patients with segmental defects and 1 with an acute fracture after external fixation. This study also included arthrodesis nails, which was beyond the scope of this study. It was impossible to extract data of the patients relevant to this study. Results are based on entire cohort. A total of three patients were lost to follow-up for bone-healing evaluation and were therefore omitted (Thonse and Conway, 2008). ^16^ This study included two patients with a pathological fracture. Data outcome of these patients cannot be extracted separately and is therefore included in the reported outcome (Kumar and Shankar, 2023). ^17^ In this study, patients received IV antibiotics 3 d prior to infection surgery. All intra-operative cultures were taken under antibiotics. Four patients had bone union after using an intramedullary nail and did not require a second stage. If the fracture was unstable with a coated implant, an external fixator was added. Planned implant removal was performed between 35 and 123 d (Qiang et al., 2007a). ^18^ Bacterial cultures were obtained from wound secretions. IV antibiotic therapy started 7–10 d preoperatively. ^19^ Only the patients with positive cultures were included. ^20^ All studies conducted on PMMA-coated implants focused on confirmed or suspected cases of infection.

### 3.3 Treatment

A total of 845 patients were treated using a single-stage approach, and 140 patients were treated with a two-stage approach. A total of 27 studies were conducted using a single-stage approach (Amorosa et al., 2013; Amorosa et al., 2014; Arsoy et al., 2018; Bakshi et al., 2022; Bhatia et al., 2017; Bidolegui et al., 2022; Charan et al., 2023; Chavan et al., 2019; Conway et al., 2014; Dar et al., 2017; Gao et al., 2019; Hackl et al., 2024; Jokhio et al., 2021; Klemm, 1986; Manikumar and Pardhasaradhim, 2024; Pradhan et al., 2017; Prasarn et al., 2009; Reddy et al., 2023; Rice et al., 2021; Saravanan et al., 2017; Shah et al., 2009; Solanki et al., 2023; Thonse and Conway, 2008; Unsworth et al., 2024; Wagner et al., 2024b; Wu and Chen, 2003), 5 studies employed a two-stage approach (Klemm and Hess, 1995; Qiang et al., 2007a; Reilly et al., 2016; Wang, 2011; Wu et al., 2007), and an additional 3 studies included patients who underwent either a single-stage or a two-stage procedure (Babhulkar et al., 2005; Kumar and Shankar, 2023; Zalikha et al., 2023). A total of 12 studies addressed the management of FRI with a conventional implant (Amorosa et al., 2013; Amorosa et al., 2014; Arsoy et al., 2018; Babhulkar et al., 2005; Hackl et al., 2024; Klemm, 1986; Prasarn et al., 2009; Shah et al., 2009; Unsworth et al., 2024; Wagner et al., 2024b; Wu and Chen, 2003; Wu et al., 2007), while 23 studies focused on the management of FRI with a custom-made PMMA-coated implant (Bakshi et al., 2022; Bhatia et al., 2017; Bidolegui et al., 2022; Charan et al., 2023; Chavan et al., 2019; Conway et al., 2014; Dar et al., 2017; Gao et al., 2019; Jokhio et al., 2021; Klemm and Hess, 1995; Kumar and Shankar, 2023; Manikumar and Pardhasaradhim, 2024; Pradhan et al., 2017; Qiang et al., 2007a; Reddy et al., 2023; Reilly et al., 2016; Rice et al., 2021; Saravanan et al., 2017; Selhi et al., 2012; Solanki et al., 2023; Thonse and Conway, 2008; Wang, 2011; Zalikha et al., 2023). Of the 12 studies on revision with a conventional implant, 10 studies used an intramedullary nail or a plate for final fracture fixation (Amorosa et al., 2013; Amorosa et al., 2014; Arsoy et al., 2018; Hackl et al., 2024; Klemm, 1986; Prasarn et al., 2009; Shah et al., 2009; Wagner et al., 2024b; Wu and Chen, 2003; Wu et al., 2007); the remaining two studies did not specify the implant used for definitive fracture fixation (Babhulkar et al., 2005; Unsworth et al., 2024). Of the 23 studies focusing on revision with a custom-made PMMA-coated implant, 11 studies used a custom-made PMMA-coated conventional implant (e.g., interlocking intramedullary nail, plate) (Bakshi et al., 2022; Bidolegui et al., 2022; Conway et al., 2014; Manikumar and Pardhasaradhim, 2024; Rice et al., 2021; Saravanan et al., 2017; Solanki et al., 2023; Thonse and Conway, 2008; Zalikha et al., 2023; Kumar and Shankar, 2023; Klemm and Hess, 1995), and 12 studies used a custom-made PMMA-coated non-conventional implant (e.g., K wires, guide wires, threaded Ilizarov rods) (Bhatia et al., 2017; Charan et al., 2023; Chavan et al., 2019; Dar et al., 2017; Gao et al., 2019; Jokhio et al., 2021; Pradhan et al., 2017; Qiang et al., 2007a; Reddy et al., 2023; Reilly et al., 2016; Selhi et al., 2012; Wang, 2011). Most studies included fracture non-union cases, which were typically defined as fractures that were not healed 6 to 9 months after the initial fracture treatment. Furthermore, many studies did not mention the exact time between fracture fixation and onset of FR. Finally, only 34 out of all 985 patients were treated for FRI within 6 months after primary fracture fixation.

### 3.4 Outcome

#### 3.4.1 Single-stage

As shown in Table 2, the mean bone-healing rate after a single-stage protocol was 80 % (range: 48 %–100 %), and the infection eradication rate was 87 % (range: 56 %–100 %). A secondary procedure was necessary in approximately 28 % of the patients (range: 7 %–100 %). The large majority of studies focused on the treatment of infected fracture non-unions (after 6 to 9 months or without mentioning a time interval between fracture fixation and onset of FRI). Only in two studies did the authors describe the inclusion of a total of six patients who were treated for FRI with a single-stage approach within 6 months of the initial fracture fixation (Wu and Chen, 2003; Pradhan et al., 2017).

**Table 2:** The mean bone-healing rate and infection eradication rate following single-stage and two-stage revision.

	Bone healing	Infection	Secondary
	N (%)	eradication N (%)	procedures N (%)
Single-stage	705/881 (80.0)	964/1109 (86.9)	328/1173 (28.0)
Conventional implants	374/465 (80.4)	611/694 (88.0)	224/709 (31.6)
Custom-made PMMA-coated implants	331/416 (79.6)	353/415 (85.0)	104/464 (22.4)
Conventional	215/257 (83.7)	226/267 (84.6)	62/233 (26.6)
Non-conventional	116/159 (73.0)	127/148 (85.8)	42/165 (25.5)
Presumed aseptic fracture non-unions	69/99 (70.0)	61/76 (80.3)	30/99 (30.3)
Two-stage	140/181 (77.3)	147/181 (81.2)	36/142 (25.4)
Conventional implants	14/24 (58.3)	14/24 (58.3)	10/15 (67.0)
Custom-made PMMA-coated implants	126/157 (80.3)	133/157 (84.7)	26/127 (20.5)
Conventional	72/86 (83.7)	73/86 (84.9)	16/86 (18.6)
Non-conventional	54/71 (76.0)	60/71 (84.5)	10/41 (24.4)

The means are an approximation based on the results described per study. In cases where studies did not explicitly describe the outcomes of patients relevant to the present study, the available outcomes of the (sub)cohort with the closest resemblance to the population of interest were reported.

**Figure 2:**
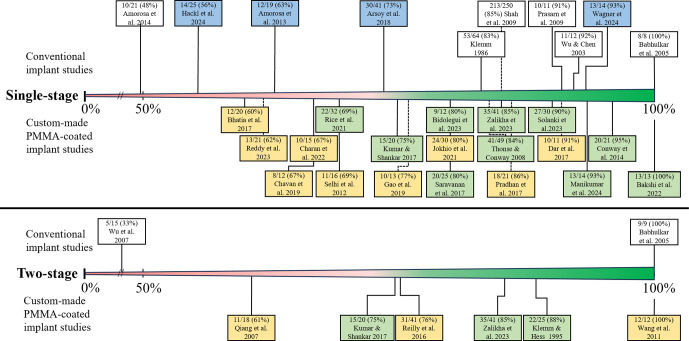
Bone-healing rate per study. In blue: the studies on patients with a presumed aseptic fracture non-union; in yellow: the studies that used custom-made PMMA-coated non-conventional implants; in green: the studies that used custom-made PMMA-coated conventional implants.

**Figure 3:**
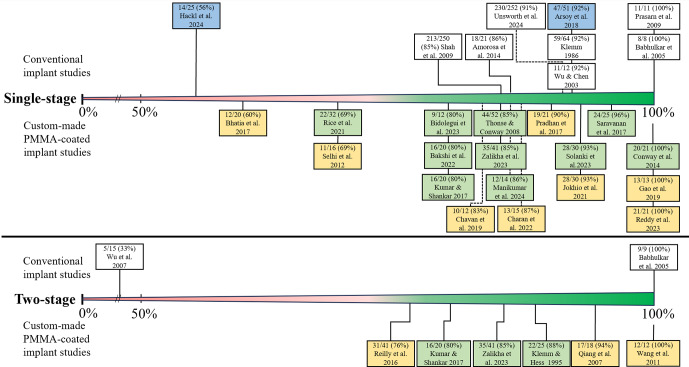
Infection eradication rate per study. In blue: the studies on patients with a presumed aseptic fracture non-union; in yellow: the studies that used PMMA-coated non-conventional implants; in green: the studies that used PMMA-coated conventional implants.

- *Conventional implant.* In patients treated with a conventional implant, the mean bone-healing rate was 80 % (Table 2) and ranged from 48 % to 100 % (Fig. 2) (Amorosa et al., 2013; Amorosa et al., 2014; Arsoy et al., 2018; Babhulkar et al., 2005; Hackl et al., 2024; Prasarn et al., 2009; Shah et al., 2009; Wagner et al., 2024b; Wu and Chen, 2003; Klemm, 1986), and the mean infection eradication rate was 88 % and ranged from 56 % to 100 % (Fig. 3) (Amorosa et al., 2014; Arsoy et al., 2018; Babhulkar et al., 2005; Hackl et al., 2024; Klemm, 1986; Prasarn et al., 2009; Shah et al., 2009; Unsworth et al., 2024; Wu and Chen, 2003). The bone-healing rate and infection eradication rate per study can also be seen in the forest plot in the Supplement (Fig. S1). Approximately 22 % of patients treated with a conventional implant required a secondary procedure (range: 7 %–64 %) (Amorosa et al., 2013; Amorosa et al., 2014; Arsoy et al., 2018; Klemm, 1986; Prasarn et al., 2009; Shah et al., 2009; Unsworth et al., 2024; Wagner et al., 2024b; Wu and Chen, 2003; Hackl et al., 2024).
- *Custom-made PMMA-coated implant.* In patients treated with a custom-made PMMA-coated implant, the mean bone-healing rate was found to be 80 %, while the mean infection eradication rate was 85 % (Table 2) (Bakshi et al., 2022; Bhatia et al., 2017; Bidolegui et al., 2022; Charan et al., 2023; Chavan et al., 2019; Conway et al., 2014; Dar et al., 2017; Gao et al., 2019; Jokhio et al., 2021; Kumar and Shankar, 2023; Manikumar and Pardhasaradhim, 2024; Pradhan et al., 2017; Reddy et al., 2023; Rice et al., 2021; Saravanan et al., 2017; Selhi et al., 2012; Solanki et al., 2023; Thonse and Conway, 2008; Zalikha et al., 2023). The range for both rates was between 60 % and 100 % (Figs. 2 and 3). A secondary procedure was required in 22 % (range: 5 %–100 %) of patients treated with a custom-made PMMA-coated implant. The mean bone-healing rate of patients treated with a custom-made PMMA-coated conventional implant was 84 % (range: 69 %–100 %) (Bakshi et al., 2022; Bidolegui et al., 2022; Conway et al., 2014; Kumar and Shankar, 2023; Manikumar and Pardhasaradhim, 2024; Rice et al., 2021; Saravanan et al., 2017; Solanki et al., 2023; Thonse and Conway, 2008; Zalikha et al., 2023), and the mean infection eradication rate was 85 % (range: 69 %–100 %) (Bakshi et al., 2022; Bidolegui et al., 2022; Conway et al., 2014; Kumar and Shankar, 2023; Manikumar and Pardhasaradhim, 2024; Rice et al., 2021; Saravanan et al., 2017; Solanki et al., 2023; Thonse and Conway, 2008; Zalikha et al., 2023). For patients treated with custom-made PMMA-coated non-conventional implants, the mean bone-healing rate was 73 % (range: 60 %–91 %) (Bhatia et al., 2017; Charan et al., 2023; Chavan et al., 2019; Dar et al., 2017; Gao et al., 2019; Jokhio et al., 2021; Pradhan et al., 2017; Reddy et al., 2023; Selhi et al., 2012), and the mean infection eradication rate was 86 % (range: 60 %–100 %) (Bhatia et al., 2017; Charan et al., 2023; Chavan et al., 2019; Gao et al., 2019; Jokhio et al., 2021; Pradhan et al., 2017; Reddy et al., 2023; Selhi et al., 2012).
- *Presumed aseptic fracture non-unions.* A total of four studies that focused on presumed aseptic fracture non-unions were included, incorporating a sample of 99 patients who were deemed to be relevant for the analysis (Amorosa et al., 2013; Arsoy et al., 2018; Hackl et al., 2024; Wagner et al., 2024b). The rate of patients with a confirmed infection based on culture results (excluding cases with positive cultures which were considered to be caused by contamination) varied between 6 % and 43 % (Amorosa et al., 2013; Arsoy et al., 2018; Hackl et al., 2024; Wagner et al., 2024b). The mean bone-healing and infection eradication rates were 70 % (range: 56 %–93 %) (Amorosa et al., 2013; Arsoy et al., 2018; Hackl et al., 2024; Wagner et al., 2024b) and 80 % (56 %–92 %) (Arsoy et al., 2018; Hackl et al., 2024), respectively. Approximately 30 % of patients who were treated with a single-stage approach for a presumed aseptic fracture non-union required a secondary procedure for bone healing or infection control.
  Three studies compared the success rate of their single-stage approach in patients who had surprise positive cultures with patients who had a truly aseptic fracture non-union (with negative cultures, as per definition) (Amorosa et al., 2013; Hackl et al., 2024; Wagner et al., 2024b). Two studies demonstrated that, in patients with positive cultures, the need for additional surgeries to achieve bone healing was significantly higher (28 % and 44 %) compared to patients with negative cultures (6.4 % and 18 %, respectively) (Amorosa et al., 2013; Hackl et al., 2024). Another study indicated that the incidence of additional surgeries was comparable between truly aseptic cases (14 %) and cases with surprise positive cultures (7 %) (Wagner et al., 2024b).

#### 3.4.2 Two-stage

Across eight studies, a total of 140 patients treated according to a two-stage protocol were included for the analysis (Babhulkar et al., 2005; Klemm and Hess, 1995; Kumar and Shankar, 2023; Qiang et al., 2007a; Reilly et al., 2016; Wang, 2011; Wu et al., 2007; Zalikha et al., 2023). The mean bone-healing rate following a two-stage protocol was 77 % (range: 33 %–100 %), whilst the mean infection eradication rate was 81 % (range: 33 %–100 %) (Table 2) (Babhulkar et al., 2005; Klemm and Hess, 1995; Kumar and Shankar, 2023; Qiang et al., 2007a; Reilly et al., 2016; Wang, 2011; Wu et al., 2007; Zalikha et al., 2023). One-quarter of patients had a need for an additional procedure after the planned second stage (range: 12 %–67 %). Most studies focused on the treatment of infected fracture non-union. One study including 12 patients focused exclusively on a two-stage treatment protocol for FRI occurring within the 3 months after primary fracture fixation (Wang, 2011). Two other studies described the treatment of 16 patients within the first 6 months after fracture fixation (Qiang et al., 2007a; Wu et al., 2007). All other studies included patients who were treated for FRI of an unhealed fracture at least 6 months after fracture fixation.

- *Conventional implant.* In two studies, including 24 patients, a conventional implant for two-stage treatment was used, as shown in Figs. 2 and 3 (Babhulkar et al., 2005; Wu et al., 2007). Both the bone-healing rate and infection eradication rate ranged from 33 % to 100 %, with a mean of 58 %. One study described the need for additional surgery after the second stage, which was required in 10 out of 15 patients (67 %) (Wu et al., 2007).
- *Custom-made PMMA-coated implant.* In six studies, comprising 116 patients who received a custom-made PMMA-coated implant, the average bone-healing rate was 80 %, with a range of 61 % to 100 %. The average infection eradication rate was 85 %, with a range of 76 % to 100 % (Klemm and Hess, 1995; Kumar and Shankar, 2023; Qiang et al., 2007a; Reilly et al., 2016; Wang, 2011; Zalikha et al., 2023). In patients treated with a custom-made PMMA-coated implant, the mean proportion of secondary procedures was 21 % (range: 12 % to 35 %). The mean bone-healing rate of patients treated with a custom-made PMMA-coated conventional implant was 84 % (range: 75 %–88 %), and the mean infection eradication was 85 % (range: 80 %–88 %) (Klemm and Hess, 1995; Kumar and Shankar, 2023; Zalikha et al., 2023). For patients treated with a PMMA-coated non-conventional implant, the mean bone healing rate was 76 % (range: 61 %–100 %), whilst the infection eradication rate was 85 % (range: 76 %–100 %) (Qiang et al., 2007a; Reilly et al., 2016; Wang, 2011).

## 4 Discussion

This systematic review evaluated the current evidence regarding the clinical outcome of single- and two-stage revision surgery for long-bone (i.e., femur, tibia, and humerus) FRI. Studies solely focused on patients with unhealed fractures without critical-sized bone defects, treated with internal fixation, were included. The mean bone-healing (80 % vs. 77 %) and infection eradication rates (87 % vs. 85 %) were similar for single-stage and two-stage revisions, though both outcomes varied significantly among studies.

### 4.1 Single- and two-stage treatment

Today, comparing the outcome of both approaches remains near to impossible. Underlying differences in anatomical locations, fracture characteristics, patient health status, and soft tissue conditions within and among studies confound any attempted comparison. Furthermore, surgical indications for both single- and two-stage approaches differed across studies. For example, in some studies, a single-stage revision was performed exclusively in patients with presumed aseptic fracture non-unions who exhibited no clinical or laboratory signs of infection (Amorosa et al., 2013; Arsoy et al., 2018; Hackl et al., 2024; Wagner et al., 2024b). In others, only cases with a strong suspicion (presence of confirmatory or suggestive signs) of infection before surgery were included (Amorosa et al., 2014; Babhulkar et al., 2005; Bakshi et al., 2022; Bhatia et al., 2017; Bidolegui et al., 2022; Charan et al., 2023; Chavan et al., 2019; Conway et al., 2014; Dar et al., 2017; Gao et al., 2019; Jokhio et al., 2021; Klemm, 1986; Kumar and Shankar, 2023; Manikumar and Pardhasaradhim, 2024; Pradhan et al., 2017; Prasarn et al., 2009; Reddy et al., 2023; Rice et al., 2021; Saravanan et al., 2017; Selhi et al., 2012; Shah et al., 2009; Solanki et al., 2023; Thonse and Conway, 2008; Unsworth et al., 2024; Wu and Chen, 2003; Zalikha et al., 2023).

As previously mentioned, a recently published systematic review and meta-analysis on single- and two-stage management of osteomyelitis, which included 42 studies (28 included single-stage revisions, 12 included two-stage revisions, and 2 compared both), demonstrated no significant difference in infection recurrence between single- and two-stage management (Lari et al., 2024). However, it is important to note that the aforementioned review, in contrast to our review, excluded studies which focused on unhealed fractures. This is an important distinction as healed fractures do not require refixation with internal fixation during revision surgery. Our review on single- and two-stage revisions for FRI in unhealed fractures without bone defects demonstrates that there is inadequate evidence in the literature to recommend one approach over the other. One very important reason is the limited number of studies published in the field of two-stage procedures in general. Furthermore, data on the outcome of FRI patients treated within the first 6 months after the primary fracture fixation are almost non-existent. As a consequence, developing consensus guidelines on treatment remains difficult. Therefore, more research in this field is needed to optimize our decision-making process with respect to the management of these sometimes very complex cases. In this context, we should acknowledge and account for the underlying differences in fracture, patient, and soft tissue characteristics and investigate whether a specific approach may be preferable in a specific subgroup of FRIs (e.g., sepsis). The recently proposed FRI classification will serve as an important tool in this effort (Alt et al., 2024). The execution of high-quality studies comparing both treatment modalities over time is essential to further advance our understanding and enhance our capabilities in this field.

### 4.2 Custom-made PMMA-coated implants

Antibiotic spacers or beads can be inserted during the initial surgery as a carrier for local antimicrobial delivery. Local antimicrobial treatment before insertion of new implants could be advantageous in controlling the infection (Metsemakers et al., 2020; Morgenstern et al., 2018; Zalavras et al., 2004). A substantial share of the included studies (23 out of 35) utilized a custom-made PMMA-coated implant as either a temporary or definitive implant. Of these, 12 studies, including both single- and two-stage approaches, used a custom-made PMMA-coated non-conventional implant. Despite the claims of several authors that these implants provide stability in fractured bones, the lack of a locking mechanism allows fracture motion around the implant, which can impede the healing process and prevent eradication of infection. Though statistical comparison was not possible, bone-healing rates tended to be lower with custom-made PMMA-coated non-conventional implants (mean: 73 %; range: 60 %–91 %) than with the use of custom-made PMMA-coated conventional implants (mean: 84%; range: 69 %–100 %) using a single-stage approach and 76 % (range: 61 %–100 %) versus 84 % (range: 75 %–88 %), respectively, using a two-stage approach. A substantial number of studies utilized a brace or cast to ensure stability until bone healing was complete (Bhatia et al., 2017; Charan et al., 2023; Chavan et al., 2019; Gao et al., 2019; Jokhio et al., 2021; Solanki et al., 2023; Qiang et al., 2007a; Dar et al., 2017), thereby supporting the hypothesis that non-conventional implants alone are inadequate in providing sufficient stability.

A total of 17 studies did not describe the routine removal of the implant (Bakshi et al., 2022; Bhatia et al., 2017; Bidolegui et al., 2022; Charan et al., 2023; Chavan et al., 2019; Conway et al., 2014; Gao et al., 2019; Jokhio et al., 2021; Kumar and Shankar, 2023; Manikumar and Pardhasaradhim, 2024; Pradhan et al., 2017; Reddy et al., 2023; Rice et al., 2021; Saravanan et al., 2017; Solanki et al., 2023; Thonse and Conway, 2008; Zalikha et al., 2023). Despite the potential for custom-made PMMA-coated implants to induce bacterial resistance due to sub-therapeutic antibiotic elution levels and to facilitate biofilm formation (Sabater-Martos et al., 2023; Ganta et al., 2024), there is no evidence to suggest that they are associated with a higher recurrence rate of infection (Ganta et al., 2024). Complications such as cement debonding, nail bending or breakage, or problems with nail extraction following the use of PMMA implants are rather common (Bhatia et al., 2017; Chavan et al., 2019; Charan et al., 2023; Pradhan et al., 2017; Qiang et al., 2007a; Saravanan et al., 2017; Selhi et al., 2012; Solanki et al., 2023). A systematic review on antibiotic cement coating in orthopedic surgery also reported a complication rate ranging from 5 % to 30 % (Ismat et al., 2021). The included studies on custom-made PMMA-coated implants demonstrate a lack of uniformity in the techniques employed for molding the implant. A wide variety of molding techniques were described, employing a range of different types of cores (e.g., K wires, guide wires, threaded Ilizarov rods). Furthermore, many different types and dosages of local antimicrobial agents were applied, which affects the mechanical strength of the PMMA (Lunz et al., 2022). This heterogeneity makes it almost impossible to compare the outcome of individual studies. Furthermore, an optimal technique to create these implants is currently not available.

### 4.3 Presumed aseptic fracture non-union

As stated in the FRI Consensus definition (Govaert et al., 2020; Metsemakers et al., 2018), the only indication of the presence of an infection might be a halt in the bone-healing process (fracture non-union). Some studies in this review treated patients with a single-stage protocol based on the assumption that they were not infected (aseptic fracture non-union). Although this indication is arbitrary as patients suffering from a fracture non-union should always be suspected of having an infection, the methodological quality and risk of bias assessment were better compared to most other studies. As such, the results of these studies are interesting to mention separately, particularly because these studies were the only ones that included an aseptic (control) group.

The results of two studies (Amorosa et al., 2013; Hackl et al., 2024) indicated that, when positive cultures are present, patients' outcomes are less favorable. This finding might indicate that the presence of an unrecognized infection may compromise the success of the initial fracture non-union procedure. It stresses the importance of a thorough diagnostic (i.e., tissue cultures) and treatment (i.e., debridement) pathway, even in cases of “presumed” aseptic fracture non-union. Furthermore, it is of particular importance to counsel these patients before revision surgery as the presence an infection can be associated with lower bone-healing rates (Amorosa et al., 2013; Hackl et al., 2024). A recently published systematic review, which included 21 studies, indeed demonstrated that an additional surgery was required in 22 % of fracture non-unions with positive cultures and in 6 % of fracture non-unions with negative cultures (Wagner et al., 2024a).

### 4.4 Time between initial fracture fixation and onset of infection

Willenegger and Roth (1986) divided FRI into early, delayed, and late onset of infection with respective cut-offs at 3 and 10 weeks (Willenegger and Roth, 1986). Other authors differentiated between early (acute) and late infections (chronic) with a cut-off of 6 weeks (Patzakis and Zalavras, 2005). However, the scientific evidence for a clear time-based cut-off to aid in the treatment decision-making process is scarce (Alt et al., 2024; Morgenstern et al., 2021). That said, the treatment approach for an FRI occurring within 6 months after the primary fracture fixation compared to one occurring after 6 months might differ significantly due to differences in fracture characteristics, the general health status of the patient, and the quality of the soft tissues, as outlined in the recently published FRI classification (Alt et al., 2024). Only five studies included patients ( n= 34) with FRIs within 6 months after the primary fracture fixation, and only one of these studies focused on this time frame. Therefore, our review shows that data regarding single- or two-stage implant exchange for the treatment of infections that occur within the first 6 months after initial fracture care are almost non-existent. Important to note is that unhealed fractures include acute fractures and fractures with and without healing potential (fracture non-unions). This fact introduces additional variability among the included studies as none of the authors adequately defined these fracture characteristics.

### 4.5 Limitations

There are several limitations that should be taken into account when interpreting the results of this review. We were unable to conduct a meta-analysis due to substantial methodological heterogeneity and fragmented data across the included studies despite excluding those that used a multiple-stage approach – which might be necessary for managing challenging infections (Cho et al., 2018; Rupp et al., 2022; Sancineto and Barla, 2008). Furthermore, there was a large variability in patient, fracture, and soft tissue characteristics among the included studies. The majority of studies were of poor or moderate methodological quality and suffered from small sample sizes and retrospective designs, limiting the strength of the evidence. In addition, the lack of standardized definitions for infection, infection eradication, and bone healing complicates comparisons across studies. Only seven studies used a (validated) definition of infection (Table S3), while other studies used no definition or used a description of infection (e.g., “clinical evidence of infection” or “presence of active infection”). Mainly in the studies focused on single-stage procedures was the reporting on additional operations inconsistent, which restricted the ability to assess the full burden of care for patients undergoing single-stage revisions. Several studies that described a single-stage protocol only mentioned the infection eradication rate and/or bone-healing rate after additional surgery. This would be a failure of single-stage treatment by definition and would lead to an overestimation of the success rate of single-stage procedures. Moreover, some studies assessed either only infection eradication or only bone healing. Numerous studies evaluated infection recurrence only over a brief follow-up period, which could also lead to an overestimation of the success rate of eradication of infection. The lack of standardized outcome measures has been highlighted in a recently published review by Reinert et al. (2025) and should be addressed. A total of 4 out of 12 studies that utilized conventional uncoated implants employed local antimicrobial agents. There was no standardized treatment protocol for the application of these local antimicrobials. Consequently, the impact of local antimicrobial treatment on outcomes could not be ascertained. Finally, the large majority of studies only included patients with an infection of the lower limb. This should be taken into account when extrapolating the results of this study to other anatomical sites, such as the upper extremity.

## 5 Conclusion

There is insufficient evidence in the current literature to recommend either a single-stage or two-stage approach using internal fixation for treating unhealed long-bone FRIs without critical-sized bone defects. It seems that, until today, the need for additional surgeries has remained high for both approaches. Furthermore, high-quality studies solely focusing on two-stage procedures, especially in case of FRIs occurring within the first 6 months after initial fracture care, are almost non-existent. Only five studies included patients with FRIs within 6 months after the primary fracture fixation, and only one of these studies focused exclusively on this time frame. Additionally, the identification of positive cultures during single-stage procedures for presumed aseptic fracture non-unions might be linked to poorer clinical outcomes. Finally, as the current literature is of poor quality and is heterogeneous in nature, future trials should use clear definitions for infection and outcome; should have at least 1-year follow-up; and should take underlying differences in patient, fracture, and soft tissue characteristics into account to guide surgical decision-making.

## Supplement

10.5194/jbji-10-347-2025-supplement The supplement related to this article is available online at https://doi.org/10.5194/jbji-10-347-2025-supplement.

## Data Availability

The data used to support the findings of this study are included in the article (Sect. 3, Table 1 and Supplement Table S3).
