# Supplementary material for: Single- versus two-stage revision surgery in the case of fracture-related infection: a systematic review
Source: J Bone Jt Infect. 2025 Oct 1;10(5):347–61. doi: 10.5194/jbji-10-347-2025 (PMC12626021; doi:10.5194/jbji-10-347-2025)
Supplement: The supplement related to this article is available online at https://doi.org/10.5194/jbji-10-347-2025-supplement. [file jbji-10-347-2025-supplement.pdf]

Supplement of J. Bone Joint Infect., 10, 347–361, 2025
https://doi.org/10.5194/jbji-10-347-2025-supplement
© Author(s) 2025. CC BY 4.0 License.

Supplement of

Single- versus two-stage revision surgery in the case of fracture-related infection: a systematic review

Jonathan Sliepen et al.

Correspondence to: Willem-Jan Metsemakers (willem-jan.metsemakers@uzleuven.be)

The copyright of individual parts of the supplement might differ from the article licence.

Table S1 Search string per database.

Source	Search string
**Pubmed**	(1 OR (2 AND 3)) AND 4<br>*Concept 1:* "IAFF"[tiab] OR "osteomyelitis"[Mesh:NoExp] OR osteomyelit*[tiab]<br>*Concept 2 AND 3:* ("Infections"[Mesh:NoExp] OR "infect*"[tiab] OR "Bone Diseases, Infectious"[Mesh:NoExp] OR "surgical wound infection"[Mesh] OR "SSI" [tiab]) AND (nonunion[tiab] OR "non union"[tiab] OR "Pseudarthrosis"[Mesh] OR pseudoarthro* [tiab] OR pseudarthro* [tiab] OR "pseudo arthro*"[tiab] OR "Fractures, Bone"[Mesh] OR "fracture*"[tiab])<br>*Concept 4:* "Reoperation"[Mesh] OR "reoperation"[tiab] OR "surgical revision"[tiab] OR "revision surger*"[tiab] OR "single stage"[tiab] OR "one stage"[tiab] OR "1 stage"[tiab] OR "one phase"[tiab] OR "single phase"[tiab] OR "2 stage"[tiab] OR "two stage"[tiab] OR "PMMA" [tiab] OR "polymethyl methacrylate" [tiab] OR "polymethyl-methacrylate" [tiab] OR "polymethylmethacrylate" [tiab] OR "antibiotic cement rod"[tiab] OR "cement rod"[tiab] OR "interlocking intramedullary nail*"[tiab] OR "bone cement" [tiab] OR "antibiotic cement*"[tiab] OR "antibiotic nail"[tiab] OR "antibiotic impregnated*" [tiab] OR "antibiotic-impregnated*" [tiab] OR "nail cement" [tiab]
**Embase**	#1 'fracture infection'/exp OR 'posttraumatic osteomyelitis'/exp OR 'IAFF':ti,ab,kw OR 'osteomyelitis'/de OR 'osteomyelit*':ti,ab,kw<br>#2 ('infection'/de OR 'infect*':ti,ab,kw OR 'bone infection'/de OR 'surgical infection'/exp OR 'SSI':ti,ab,kw) AND ('fracture nonunion'/exp OR nonunion:ti,ab,kw OR 'non union':ti,ab,kw OR 'pseudarthrosis'/de OR 'pseudoarthro*':ti,ab,kw OR 'pseudarthro*':ti,ab,kw OR 'pseudo arthro*':ti,ab,kw OR 'fracture'/exp OR 'fracture*':ti,ab,kw)<br>#3: #1 OR #2<br>#4 'reoperation'/exp OR 'reoperation':ti,ab,kw OR 'revision surgery'/exp OR 'surgical revision':ti,ab,kw OR 'revision surger*':ti,ab,kw OR 'one-stage revision'/exp OR 'single stage':ti,ab,kw OR 'one stage':ti,ab,kw OR '1 stage':ti,ab,kw OR 'one phase':ti,ab,kw OR 'single phase':ti,ab,kw OR 'two-stage revision'/exp OR '2 stage*':ti,ab,kw OR 'two stage*':ti,ab,kw OR 'PMMA':ti,ab,kw OR 'polymethyl methacrylate':ti,ab,kw OR 'polymethyl-methacrylate':ti,ab,kw OR 'polymethylmethacrylate':ti,ab,kw OR 'antibiotic cement rod':ti,ab,kw OR 'cement rod':ti,ab,kw OR 'interlocking intramedullary nail*':ti,ab,kw OR 'bone cement':ti,ab,kw OR 'antibiotic cement*':ti,ab,kw OR 'antibiotic nail':ti,ab,kw OR 'antibiotic impregnated*':ti,ab,kw OR 'antibiotic-impregnated*':ti,ab,kw OR 'nail cement':ti,ab,kw<br>#5: #3 AND #4<br>NOT ('conference abstract':it OR preprint:it)
**Web of science Core Collection**	**Science Citation Index Expanded (SCI-EXPANDED)--1955-present**<br>**Social Sciences Citation Index (SSCI)--1956-present**<br>**Arts & Humanities Citation Index (AHCI)--1975-present**<br>**Conference Proceedings Citation Index – Science (CPCI-S)--1990-present**<br>**Emerging Sources Citation Index (ESCI)--2017-present**<br><br>**Concept 1** TS=("IAFF" OR osteomyelit*)<br>**Concept 2 AND 3** TS=(("infect*" OR "SSI") AND ("nonunion" OR "non union" OR pseudoarthro* OR pseudarthro* OR "pseudo arthro*" OR "fracture*"))<br>**Concept 4** TS=("reoperation" OR "surgical revision" OR "revision surger*" OR "single stage" OR "one stage" OR "1 stage" OR "one phase" OR "single phase" OR "2 stage" OR "two stage" OR "PMMA" OR "polymethyl methacrylate" OR "polymethyl-methacrylate" OR "polymethylmethacrylate" OR "antibiotic cement rod" OR "cement rod" OR "interlocking intramedullary nail*" OR "bone cement" OR "antibiotic cement*" OR "antibiotic nail" OR "antibiotic impregnated*" OR "antibiotic-impregnated*" OR "nail cement")
**Grey literature**	
**Cochrane Library (CENTRAL)**	#1 ([mh ^"osteomyelitis"] OR ("IAFF" OR osteomyelit*):ti,ab,kw)<br>#2 ([mh ^"Infections"] OR [mh ^"Bone Diseases, Infectious"] OR [mh "surgical wound infection"] OR (infect* OR "SSI"):ti,ab,kw)<br>AND ([mh "Pseudarthrosis"] OR [mh "Fractures, Bone"] OR (nonunion OR "non union" OR pseudoarthro* OR pseudarthro* OR (pseudo NEXT arthro*) OR fracture*):ti,ab,kw)<br>#3: #1 OR #2<br>#4 [mh "Reoperation"] OR ("reoperation" OR "surgical revision" OR (revision NEXT surger*) OR "single stage" OR "one stage" OR "1 stage" OR "one phase" OR "single phase" OR "2 stage" OR "two stage" OR "PMMA" OR "polymethyl methacrylate" OR "polymethyl-methacrylate" OR "polymethylmethacrylate" OR "antibiotic cement rod" OR "cement rod" OR "interlocking intramedullary nail*" OR "bone cement" OR "antibiotic cement*" OR "antibiotic nail" OR "antibiotic impregnated*" OR "antibiotic-impregnated*" OR "nail cement"):ti,ab,kw<br>#5: #3 AND #4
**ICTRP**	Fracture-related infection<br>Infected non-union

Table S2 Quality assessment using the McMaster critical appraisal.

Categories	Amorosa et al. (2013)	Arsoy et al. (2018)	Hackl et al. (2024)	Wagner et al. (2024)	Prasarn et al. (2009)	Amorosa et al. (2014)	Klemm (1986)	Shah et al. (2009)	Unsworth et al. (2024)	Wu and Chen (2003)	Babhulkar et al. (2005)	Wu et al. (2007)	Bakshi et al. (2022)	Bhatia et al (2017)	Bidolegui et al. (2023)	Charan et al. (2022)	Chavan et al. (2019)	Conway et al. (2014)
**1. Study purpose**																		
Was the study purpose clearly stated?	1	1	1	1	1	1	0	0	1	1	0	1	1	1	0	1	1	1
**2. Literature review**																		
Was relevant background literature reviewed?	1	1	1	1	1	1	0	1	1	1	1	1	1	1	1	1	1	1
**3. Study design**	RCS	RCS	RCS	RCS	CS	RCS	CS	CS	RCS	CS	RCS	RCS	RPS	CS	RCS	CS	CS	RCS
**4. Sample**																		
Was the sample described in detail?	1	1	1	1	1	0	0	1	0	1	0	1	1	1	1	0	0	0
Was the sample justified?	1	1	1	1	1	1	0	1	1	0	1	1	0	0	0	0	0	1
Were the groups randomized?	NA	NA	NA	NA	NA	NA	NA	NA	NA	NA	NA	NA	1	NA	NA	NA	NA	NA
Was randomizing appropriate done?	NA	NA	NA	NA	NA	NA	NA	NA	NA	NA	NA	NA	0	NA	NA	NA	NA	NA
**5. Outcomes**																		
Were the outcome measures reliable?	1	1	0	1	0	0	0	0	1	0	0	0	0	0	1	0	0	0
Were the outcome measures valid?	1	1	1	1	1	1	1	1	1	1	1	1	0	1	0	1	0	1
**6. Intervention**																		
Intervention was described in detail?	1	1	1	0	1	1	1	1	1	1	1	1	1	1	1	1	1	1
Contamination was avoided?	1	1	1	1	NA	1	NA	NA	1	NA	NA	NA	1	0	0	NA	NA	1
Cointervention was avoided?	0	0	1	0	0	1	NA	NA	0	0	NA	NA	0	1	0	NA	NA	1
**7. Results**																		
Results were reported in terms of statistical significance?	1	1	1	1	0	1	0	0	1	0	0	1	1	0	0	0	0	0
Were the analysis method(s) appropriate?	1	1	1	1	1	1	1	1	1	1	1	1	1	1	1	0	0	1
Clinical importance was reported?	1	1	1	0	0	1	1	1	1	1	1	0	0	1	1	0	0	1
Drop-outs were reported?	1	1	1	1	1	1	0	1	1	1	1	1	0	0	0	0	0	1
**8. Conclusions and implications**																		
Conclusions were appropriate given study methods and results?	1	1	1	1	1	1	0	1	1	1	1	1	1	1	1	1	0	1
**Total points**	13	13	13	11	9	12	4	9	12	9	8	10	9	9	7	5	3	11
**Max points**	14	14	14	14	13	14	12	12	14	13	12	12	16	14	14	12	12	14
**Score (%)**	93%	93%	93%	79%	69%	86%	42%	33%	86%	69%	67%	83%	56%	64%	50%	42%	25%	79%
**Study quality interpretation**	E	E	E	G	M	G	P	P	G	M	M	G	M	M	M	P	P	G

NA: Not applicable; CS: Case series; RCS: retrospective cohort study; PCS prospective cohort study; RPS: randomised prospective study. E: excellent; G: good; M: moderate; P:poor

Categories	Dar et al. (2017)	Gao et al. (2019)	Jokhio et al. (2021)	Manikumar et al. (2024)	Pradhan et al. (2017)	Reddy et al. (2023)	Rice et al. (2021)	Saravanan et al. (2017)	Selhi et al. (2012)	Solanki et al. (2023)	Thonse and Conway (2008)	Kumar and Shankar (2023)	Zalikha et al. (2023)	Klemm and Hess (1995)	Qiang et al. (2007)	Reilly et al. (2016)	Wang (2011)
**1. Study purpose**																	
Was the study purpose clearly stated?	0	0	0	1	1	1	0	0	1	1	0	1	1	0	1	1	1
**2. Literature review**																	
Was relevant background literature reviewed?	1	1	1	0	1	1	1	1	1	1	1	1	1	0	1	1	0
**3. Study design**	CS	CS	CS	CS	CS	RCS	CS	CS	CS	CS	RCS	CS	RCS	CS	CS	RCS	CS
**4. Sample**																	
Was the sample described in detail?	1	1	1	1	1	0	1	1	1	1	1	1	1	1	1	1	1
Was the sample justified?	1	0	0	0	1	1	0	0	0	1	1	0	1	0	0	1	1
Were the groups randomized?	NA	NA	NA	NA	NA	NA	NA	NA	NA	NA	NA	NA	NA	NA	NA	NA	NA
Was randomizing appropriate done?	NA	NA	NA	NA	NA	NA	NA	NA	NA	NA	NA	NA	NA	NA	NA	NA	NA
**5. Outcomes**																	
Were the outcome measures reliable?	0	0	0	0	0	0	0	0	0	0	0	1	1	0	0	1	0
Were the outcome measures valid?	1	0	1	1	1	0	1	1	1	1	1	0	1	1	0	1	1
**6. Intervention**																	
Intervention was described in detail?	1	1	1	1	1	1	1	1	1	1	1	1	1	1	1	1	1
Contamination was avoided?	NA	NA	NA	NA	NA	NA	NA	NA	NA	NA	0	0	0	0	NA	0	NA
Cointervention was avoided?	NA	NA	NA	NA	NA	NA	NA	NA	NA	NA	1	0	1	0	NA	0	NA
**7. Results**																	
Results were reported in terms of statistical significance?	0	0	0	0	0	0	0	0	0	0	0	1	1	0	0	1	0
Were the analysis method(s) appropriate?	0	1	0	0	1	0	0	0	1	0	1	1	1	0	1	1	1
Clinical importance was reported?	0	1	1	1	1	0	0	0	1	1	1	1	1	1	1	1	1
Drop-outs were reported?	0	0	0	0	1	0	0	0	0	0	1	0	1	0	0	1	0
**8. Conclusions and implications**																	
Conclusions were appropriate given study methods and results?	0	1	0	0	1	1	1	0	1	1	1	1	1	0	1	1	1
**Total points**	5	6	5	5	10	5	5	4	8	8	10	9	13	4	7	12	8
**Max points**	12	12	12	12	12	12	12	12	12	12	14	14	14	14	12	14	12
**Score (%)**	42%	50%	42%	42%	83%	42%	42%	33%	67%	67%	71%	64%	93%	29%	58%	86%	67%
**Study quality interpretation**	P	M	P	P	G	P	P	P	M	M	M	M	E	P	M	G	M

NA: Not applicable; CS: Case series; RCS: retrospective cohort study; PCS prospective cohort study; RPS: randomised prospective study. E: excellent; G: good; M: moderate; P:poor

Table S3 Additional data.

**Author** (year of publication)	**Country of publication**	**Definition of infection**	**Age in years** mean (range)	**Initial implant**	**Implant at revision**	**Coated implant**	**Core of implant**	**Local antimicrobials**	**Bone graft**	**Pathogens cultured**
**Studies on presumed aseptic non-union***										
Amorosa et al. (2013)	USA	No	Not specified	Not specified	Plate or IMN	No	-	-	-	*C. acnes* 3x, CoNS 17x, MSSA 1x, Mixed flora 2x
Arsoy et al. (2018)	USA	No	47 (18-96)[1]	Not specified	Plate or IMN	No	-	-	50/51 (Autograft; allograft; biocomposite; bone morphogenic protein; demineralized bone matrix)	*C. acnes*, CoNS, *S. epidermidis*, *S. aureus*, *S. saccharolyticus*, *S. intermedius*, *Strep. viridans*
Hackl et al. (2024)	Germany	Yes	43 (18-74)	Femoral nail (n=25)	Interlocking nail	No	-	-	-	*S. epidermidis* 10x, *S. capitis* 3x, *S. lugdunensis* 3x, *S. haemolyticus* 2x, *S. warneri* 2x, *S. hominis* 1x, *S. aureus* 1x, *Streptococcus alactolyticus* 1x, *Enterococcus faecalis* 2x, *Pseudomonas aeruginosa* 1x, *Pseudomonas fluorescenses* 1x, *C. acnes* 2x
Wagner et al. (2024)	The Netherlands	Yes	* 53 (not specified)	Not specified	Intramedullary nail / plate	No	-	-	-	CoNS 10x, *C. acnes* 8x, mixed flora 4x
**Studies with confirmed or suspected infection**										
Prasarn et al. (2009)	USA	No	53	Plate (n=8) IMN (n=3)	Dynamic condylar screw plate (n=4) Blade plate (n=5) IMN (n=2)	No	-	-	ICBG (n=2), Demineralized bone matrix (n=3), None (n=6)	*S. epidermidis* 4x, Mixed flora 2x, MRSA 2x, *Enterococcus faecalis*, Culture negative 2x
Amorosa et al. (2014)	USA	No	Not specified	Not specified	95-degree angled blade plate	No	-	-	ICBG/Demineralized bone matrix	Not specified
Klemm (1986)	Germany	No	27 (not specified)	Plate (n=28) IMN (n=36)	IMN	No	-	-	-	Not specified
Shah et al. (2009)	Nepal	Yes	Not specified	Not specified	IMN (SIGN)	No	-	-	-	Not specified
Unsworth et al. (2024)	United Kingdom	Yes	Group 1: median 57 (IQR: 40-66) Group 2: median 52 (IQR: 37-62)	Not specified	Not specified	No	Not specified	Vancomycin/ gentamicin/ tobramycin or combination	-	Not specified
Wu and Chen (2003)	Taiwan	No	Median: 37 (18-72)	Sliding hip screw	Sliding hip screw	No	-	Vancomycin (+ gentamycin)	CBG from medial tibial condyle	MRSA 8x, MSSA 1x, *Pseudomonas aeruginosa* 2x, Mixed flora 2x
Babhulkar et al. (2005)	India	No	Not specified	Not specified	Not specified	Yes, (n=4)	Interlocking IMN	Gentamycin	Yes, (n=6) not specified	Not specified
Wu et al. (2007)	Taiwan	No	48 (16-82)	Sliding hip screw (n=22) Gamma nail (n=1)	1st stage: antibiotic loaded beads + external fixation or buck traction 2nd stage: plating	No	-	Not specified	CBG	MRSA 4x, MSSA 4x, Enterococcus spp. 2x, *Pseudomonas aeruginosa* 1x, *Enterobacter cloacae* 1x, Acinetobacter 1x, Mixed flora 8x, Culture negative 2x
**PMMA-coated implant studies****										
Bakshi et al. (2022)	India	No	Not specified (21-40)	Plate (n=5)) IMN (n=8)	Cement coated interlocking IMN	Yes	Interlocking IMN	Vancomycin (+ gentamycin)	Yes, (n=7) not specified	*S. aureus* 15x, *Pseudomonas aeruginosa* 2x, E. coli 1x, Mixed flora 2x
Bhatia et al. (2017)	India	No	39 (22-61)	Not specified	Cement coated K-nail	Yes	K-nail	Vancomycin + teicoplanin	-	*S. aureus* 12x, culture negative 8x
Bidolegui et al. (2023)	Brasil	No	53 (24-72)	Plate (n=12) IMN (n=3)	Cement coated plate	Yes	Plate	Vancomycin (+ gentamycin)	Yes, not specified	MSSA 8x, *Enterobacter cloacae* 2x, *Proteus mirabilis* 1x, *Streptococcus viridans* 1x, *E. coli* 1x, Culture negative 2x

Charan et al. (2022)	India	No	Not specified	Not specified	Cement coated interlocking IMN	Yes	Interlocking IMN	Vancomycin	-	Not specified
Chavan et al. (2019)	India	No	Not specified	Not specified	Cement coated K-nail	Yes	K-nail	Vancomycin + clindamycin	-	Not specified
Conway et al. (2014)	USA	No	37 (15-74)[1]	Not specified	Cement coated intramedullary locking rod	Yes	Locking rod	Vancomycin + tobramycin	-	MRSA in 15x, Mixed flora 18x, Culture negative 10x
Dar et al. (2017)	India	No	34 (21-64)	Not specified	Cement coated threaded Ilizarov rod	Yes	Threaded Ilizarov rod	Tobramycin (5x) Gentamycin + Vancomycin (2x) Gentamycin (2x) Amikacin (2x)	-	*S. aureus* 5x, *E. coli* 2x, Pseudomonas spp. 1x, Mixed flora 1x, Culture negative 2x
Gao et al. (2019)	China	No	45 (19-79)	IMN (n=4) Plate (n=7)	Cement coated k-wire or pedicle	Yes	K-wire or pedicle	Vancomycin + gentamicin	-	*S. aureus* 7x, *Acinetobacter baumannii* 1x, *Enterobacter aerogenes* 1x, *S. haemoliticus* 1x, culture negative 3x
Jokhio et al. (2021)	Pakistan	No	Not specified (22-61)	IMN	Cement coated K-nail	Yes	K-nail	Vancomycin + Teicoplanin	-	*S. aureus* 16x Not further specified
Manikumar et al. (2024)	India	No	Not specified (20-70)	Not specified	Cement coated interlocking IMN	Yes	Interlocking IMN	Vancomycin	-	Not specified
Pradhan et al. (2017)	India	No	34 (20-63)	IMN (n=18) Plate (n=2) External fixator (n=1)	Cement coated K-nail	Yes	K-nail	Vancomycin + gentamycin	-	MSSA: 10x, MRSA: 5x, CoNS 1x, Enterobacter 1x, *Pseudomonas aeruginosa* 1x, Klebsiella 1x, Mixed flora 1x, Culture negative 1x
Reddy et al. (2023)	India	No	43 (18-72)	Not specified	Cement coated Ender's nail/titanium IMN	Yes	Cement coated Ender's nail/ titanium IMN	Vancomycin + gentamicin	-	Not specified
Rice et al. (2021)	USA	Yes	47 (not specified)	Not specified	Cement coated interlocking IMN	Yes	Interlocking IMN	Vancomycin + tobramycin, + gentamicin	-	Not specified
Saravanan et al. (2017)	India	No	Not specified (21-65)	Not specified	Cement coated locked K-nail	Yes	Locked K-nail		-	Not specified
Selhi et al. (2012)	India	No	39 (18-54)	IMN (n=8) External fixator (n=5) Plate (n=3)	Cement coated K-nail, guidewire, interlocking IMN	Yes	K-nail (n=11) Guidewire (n=4) Interlocking IMN (n=1)	Vancomycin + gentamycin	-	*S. aureus* 5x, Enterobacter 3x, Culture negative 8x
Solanki et al. (2023)	India	No	40 (23-67)	Not specified	Cement coated locked K-nail	Yes	Locked K-nail	Not specified	-	Not specified
Thonse and Conway (2008)	USA	No	46 (16-86)[1]	Not specified	Cement coated interlocking IMN	Yes	Interlocking IMN	Vancomycin + tobramycin	-	Not specified
Kumar and Shankar (2023)	India	No	36 (23-50)	Not specified	Polymer coated nail or cement coated interlocking IMN	Yes	Polymer coated nail or cement coated interlocking IMN	Vancomycin + gentamicin	-	MRSA 8x, Pseudomonas 1x, mixed flora 7x, culture negative 4x
Zalikha et al. (2023)	USA	Yes	39 (20-66)	IMN (n=41)	1st stage: cement coated interlocking nail 2nd stage: exchange cement coated interlocking IMN	Yes	Interlocking IMN	Vancomycin + tobramycin	-	*S. aureus* 22x, Mixed flora 9x, Culture negative 3x, Other 7x

Klemm and Hess (1995)	Germany	No	38 (17-58)	Not specified	1st stage: external fixator + gentamicin cement beads 2nd stage: interlocking IMN with cement coated surgical wire inside	Yes	Multistranded surgical wire	Gentamicin	Yes (n=12), not specified	Not specified
Qiang et al. (2007)	China	No	38 (22-78)	IMN (n=18)	1st stage: cement coated guidewire 2nd stage: Interlocking IMN	Yes	Guidewire	Vancomycin	Yes, (n=6) not specified	*S. aureus* 8x, Culture negative 11x
Reilly et al. (2016)	USA	Yes	41 (15-78)	IMN (n=41)	1st stage: cement coated guidewire or threaded Ilizarov wire 2nd stage: Interlocking IMN	Yes	Ilizarov rod or ball-tipped guidewire	Not specified	-	*S. aureus* 27x, Other 13x
Wang (2011)	China	No	40 (26-53)	IMN (n=12)	1st stage: cement coated K-wire 2nd stage: locking plate	Yes	K-wire	Vancomycin	ICBG 10x	MRSA 8x, Streptococcus spp. 2x, Culture negative 2x

C.: Cutibacterium; CBG: Cancellous bone graft; CoNS: Coagulase negative staphylococci; E.: Escherichia; ICBG: Iliac crest bone graft; IMN: intramedullary nail; K-wire: Kirschner-wire; K-nail: Küntscher nail; MRSA: methicillin resistant staphylococcus aureus; MSSA: methicillin sensitive staphylococcus aureus; S.: staphylococcus; Spp: species; UTN: unreamed tibia nail; USA: United States of America; [1] number is based on the entire population or the subpopulation closest related to the patients relevant to our study.
* Only the patients with positive cultures were included.
** All studies conducted on PMMA-coated implants were concerned with confirmed or suspected cases of infection.

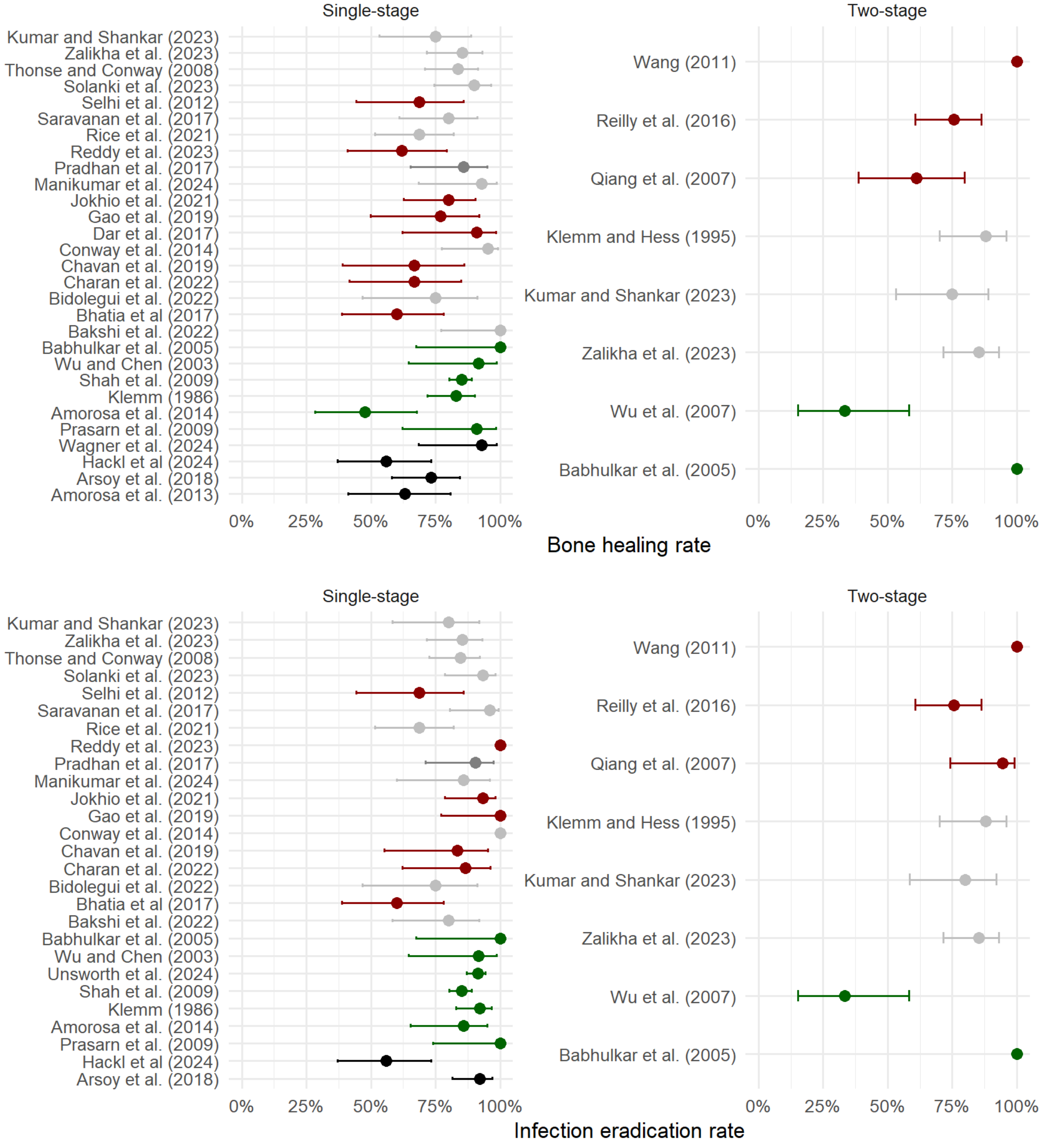

Figure S1 A forest plot displaying the bone healing rate and infection eradication rate of single-stage and two-stage revision per study. Black: studies on presumed patients with an aseptic fracture non-union; Green; studies that used conventional implants without a PMMA coating; Red: : the studies that used PMMA-coated non-conventional implants Grey: the studies that used PMMA-coated conventional implants.
